# Three-dimensional Co_3_O_4_@MWNTs nanocomposite with enhanced electrochemical performance for nonenzymatic glucose biosensors and biofuel cells

**DOI:** 10.1098/rsos.170991

**Published:** 2017-12-20

**Authors:** Kailong Jiao, Yu Jiang, Zepeng Kang, Ruiyun Peng, Shuqiang Jiao, Zongqian Hu

**Affiliations:** 1 State Key Laboratory of Advanced Metallurgy, University of Science and Technology Beijing, Beijing, 100083, People's Republic of China; 2 Beijing Institute of Radiation Medicine, Beijing, 100850, People's Republic of China; 3 Department of Orthopedics, Peking University Third Hospital, Beijing, 100191, People's Republic of China

**Keywords:** Co_3_O_4_ nanoarchitecture, multi-walled carbon nanotubes, nonenzymatic, glucose biosensor, glucose biofuel cell

## Abstract

Three-dimensional nanoarchitectures of Co_3_O_4_@multi-walled carbon nanotubes (Co_3_O_4_@MWNTs) were synthesized via a one-step process with hydrothermal growth of Co_3_O_4_ nanoparticles onto MWNTs. The structure and morphology of the Co_3_O_4_@MWNTs were characterized by X-ray diffraction, Fourier transform infrared spectroscopy, Brunauer–Emmett–Teller, scanning electron microscopy and transmission electron microscopy. The electrocatalytic mechanism of the Co_3_O_4_@MWNTs was studied by X-ray photoelectron spectroscopy and cyclic voltammetry. Co_3_O_4_@MWNTs exhibited high electrocatalytic activity towards glucose oxidation in alkaline medium and could be used in nonenzymatic electrochemical devices for glucose oxidation. The open circuit voltage of the nonenzymatic glucose/O_2_ fuel cell was 0.68 V, with a maximum power density of 0.22 mW cm^−2^ at 0.30 V. The excellent electrochemical properties, low cost, and facile preparation of Co_3_O_4_@MWNTs demonstrate the potential of strongly coupled oxide/nanocarbon hybrid as effective electrocatalyst in glucose fuel cells and biosensors.

## 1. Introduction

Glucose biofuel cells (GBFCs) have received much attention as promising next-generation energy storage systems [1–4], due to the abundance of glucose sources (e.g. starch, cellulose [5], body fluids [6]) and very high energy (−2.87 × 10^3^ kJ mol^−1^) produced after complete glucose oxidation [7]. However, the performance of GBFCs strongly depends on the catalysts, which are crucial for the glucose oxidation reaction (GOR) [1]. Generally, two kinds of catalysts are used in GBFCs: biological and non-biological [8]. Biological catalysts are specific enzymes for the anodic oxidation of glucose molecules. However, enzymes are restricted in their application to GBFCs, due to their poor stability [6], poor immobilization techniques [8], susceptibility to the operating environment and vulnerability with long-term running [9]. These problems must be overcome for the development of enzymatic GBFCs [5]. Examples of non-biological catalysts include precious metals (Pd [10] and Au [6,11,12]) and metal oxides (Co_3_O_4_ [13] and CuO [14]), which possess exceptional catalytic properties for oxidation of glucose [15–17].

Recently, cobalt oxide (Co_3_O_4_) has attracted attention as a non-biological catalyst due to its low cost and good electrochemical and catalytic properties [18–21]. A recent report described Co_3_O_4_ hollow nanododecahedra having excellent catalytic abilities towards glucose oxidation in GBFCs [13], opening the possibility of using Co_3_O_4_ as an appropriate alternative for glucose enzymes. However, the electrical conductivity of Co_3_O_4_ is poor, and conductive carbon-based supports are generally employed when exploring the catalytic performance of Co_3_O_4_.

Extensive efforts have focused on the design of metal/metal oxide and carbon composites to enhance catalytic activity. Among the explored carbon-based nanomaterials, carbon nanotubes (CNTs) with high electrical conductivity, large surface area, high mechanical strength and structural flexibility [22], are promising substrates for composite catalysts [23–25]. Metal oxide/CNTs nanocomposites, combining the advanced properties of each component, have drawn interest for use in catalysis, sensor and energy storage. The reactions occurring to multi-walled CNTs cannot destroy the inner graphitic walls of multi-walled carbon nanotubes, thus ensuring a good conducting network [22]. When Co_3_O_4_ was coupled with CNTs, it showed better performance than MWNTs, cubic spinel Co_3_O_4_, or their physical mixture [4].

**Scheme 1.**
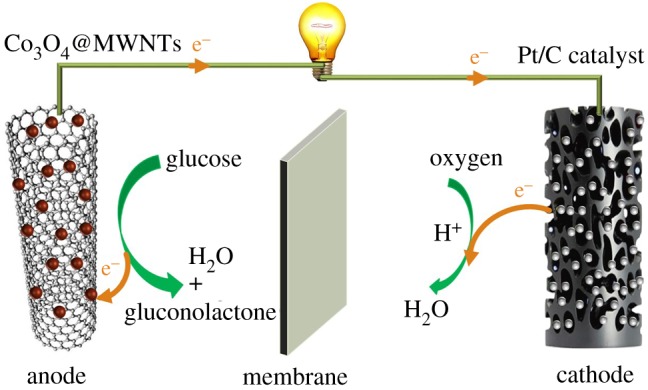
Schematic diagram of the glucose-powered fuel cell based on 3D Co_3_O_4_@MWNTs material as anode and cathode.

In this study, we constructed three-dimensional (3D) nanoarchitectures of Co_3_O_4_@MWNTs by a facile hydrothermal method. The prepared Co_3_O_4_@MWNTs nanocomposites exhibited high electrocatalytic activity and outstanding stability for glucose biosensors and glucose fuel cells (GFCs) (scheme 1).

## 2. Material and methods

### 2.1. Materials

MWNTs functionalized with carboxylic acid groups (CNTs, greater than 95%, 8–15 nm in diameter, approximately 50 µm in length, produced by the chemical vapour deposition (CVD) method) were purchased from Aladdin. NH_4_OH (25–28%) and KOH were obtained from Xilong Scientific. Alcohol, glucose and Co(Ac)_2_·4H_2_O were obtained from Sinopharm Chemical Reagent Co., Ltd, China. Nafion^®^ 117 solution was purchased from Sigma-Aldrich. The proton exchange membrane and carbon fibre sheet were obtained from Sigma-Aldrich. All chemicals and solvents were used without further purification.

### 2.2. Synthesis of samples

Co_3_O_4_@MWNTs composite was synthesized by a simple one-step hydrothermal reaction. MWNTs (0.1 g) were dispersed in 60 ml of alcohol under ultrasonication for 30 min. A certain amount of Co(Ac)_2_·4H_2_O was added to the dispersion with continued ultrasonication for 20 min. NH_4_OH and deionized water were successively added dropwise to the dispersion with ultrasonication for 10 min. The dispersion was conducted by backflow in oil at 80°C for 10 h before being transferred into a 200 ml Teflon autoclave. After hydrothermal reaction at 150°C for 3 h, black precipitate was collected by centrifugation and washed several times with deionized water and alcohol, as shown in electronic supplementary material, scheme S1. The final product was dried at 80°C for 3 h in a vacuum oven and directly used as the GOR catalyst without any further treatment. Co_3_O_4_ was synthesized with the same method but without the addition of MWNTs.

### 2.3. Apparatus measurements

Field-emission scanning electron microscopy (SEM; JEOL, JSM-6701) observation was performed. Transmission electron microscopy (TEM; JEOL, JEM-2010) and high-resolution transmission electron microscopy (HRTEM) images were obtained with an energy dispersive X-ray spectroscopy detector. X-ray diffraction (XRD) patterns were collected by using an X-ray diffractometer (Rigaku Ultima IV) with Cu K*α* radiation (*λ* = 1.5406 Å). Fourier transform infrared spectroscopy (FTIR) was measured on a 470 FTIR spectrometer. Brunauer–Emmett–Teller (BET) was conducted on a Micromeritics Instrument Corporation sorption analyser (Micromeritics TriStar II 3020). X-ray photoelectron spectroscopy (XPS) measurements were carried out on a PHI Quantera SXM model spectrometer, to obtain the composition of the materials. Binding energies reported in this study were revised by the C 1 s peak (284.8 eV).

### 2.4. Electrochemical measurements

The modified electrode was prepared through three steps. (i) The glassy carbon electrode (GCE) was polished with alumina slurry and ultrasonically cleaned in deionized water and ethanol, respectively. (ii) A 10 mg aliquot of the prepared material was dispersed in 1 ml of deionized water and ultrasonically dispersed for 10 min. (iii) A 5 μl aliquot of dispersion liquid was dropped onto the surface of the GCE. After the GCE was dried, 5 µl of Nafion solution (0.5%) was dropped onto the surface of the GCE, which was dried at room temperature. This method was also used to prepare the electrode of the GFC.

Electrochemical measurements were performed with a CHI 660E electrochemical workstation (Beijing, China). Cyclic voltammetry (CV) measurements were made in the 0–0.7 V region with an electrolyte of 0.1 M KOH, by using the conventional three-electrode system, with the modified GCE as the working electrode, Pt sheet as the counter electrode, and Ag/AgCl electrode as the reference electrode [13].

The GFC, with organic glass as the shell material, was separated into anodic and cathodic compartments by a proton exchange membrane. Solutions filling the anode and cathode chambers were argon-saturated 0.1 M KOH/0.1 M glucose solution and oxygen-saturated buffer solution, respectively. The anode was prepared by smearing a carbon fibre sheet (active area: 1 × 1 cm^2^) with the suspension liquid mentioned above. The open circuit voltage (*E*^ocv^) was measured by a digital multimeter. The power density of the fuel cell was calculated by the voltage on the variable external resistance linking the fuel cell and the current through it.

## 3. Results and discussion

### 3.1. Morphology and structure

**Figure. 1.**
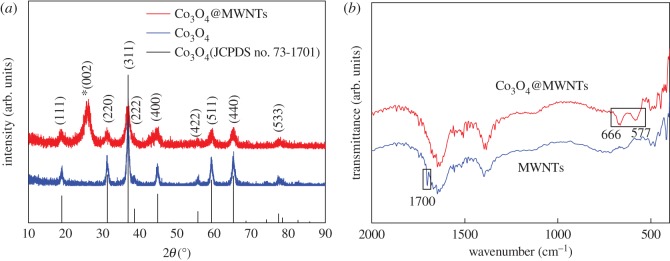
XRD pattern of the Co_3_O_4_@MWNTs and Co_3_O_4_ (*a*) and FTIR spectra for Co_3_O_4_@MWNTs and MWNTs (*b*).

Figure 1*a* shows the XRD patterns of pure Co_3_O_4_ (blue line) and Co_3_O_4_@MWNTs composite (red line). Diffraction peaks at 2*θ* = 19.0°, 31.3°, 36.8°, 38.6°, 44.8°, 55.7°, 59.4°, 65.2° and 77.3° can be well assigned to the (111), (220), (311), (222), (400), (422), (511), (440) and (533) crystal planes of the Co_3_O_4_ (JCPDS no. 73–1701) and 26.2° assigned to the (002) crystal plane of graphite (JCPDS no. 75–1621) from the red line. The Co_3_O_4_@MWNTs were considered to be synthesized successfully, because there were no other diffraction peaks observed except those of the CNTs (red line) and Co_3_O_4_ (blue line). The Co_3_O_4_ crystallites on the CNTs were smaller than the pure Co_3_O_4_ crystallites from the broader and weaker XRD peaks of the Co_3_O_4_@MWNTs compared to the peaks of the pure Co_3_O_4_ (blue line).

The FTIR spectra of Co_3_O_4_@MWNTs and MWNTs were recorded in figure 1*b* to study the formation mechanism of Co_3_O_4_. On comparison, the IR spectrum of MWNTs displays a distinct peak at 1700 cm^−1^ that originate from the stretching vibration of C = O of carboxyl, while the IR spectrum of Co_3_O_4_@MWNTs displays two distinct peaks at 577 cm^−1^ and 666 cm^−1^. The peak at 577 cm^−1^ relates to the OB3 vibration in the spinel lattice, where B denotes Co^3+^ in an octahedral hole, and the other peak at 666 cm^−1^ is connected with the ABO3 vibration, where A denotes the Co^2+^ in a tetrahedral hole [26]. When the two metal–oxygen bonds appeared, the C = O bond disappeared. Hence, we can draw the conclusion that the formation of Co_3_O_4_ takes place on carboxyl groups of the MWNTs.

The N_2_ adsorption–desorption isotherms of the Co_3_O_4_ and Co_3_O_4_@MWNTs were shown in electronic supplementary material, figure S3, in which the adsorption isotherm and desorption isotherm were superposed, demonstrating that there was no pore existing on the surface of the materials. The BET surface area of Co_3_O_4_ and Co_3_O_4_@MWNTs were 67.2 m² g^−1^ and 70.0 m² g^−1^, respectively, indicating the specific area increased after the composition of Co_3_O_4_ and MWNTs.

**Figure 2.**
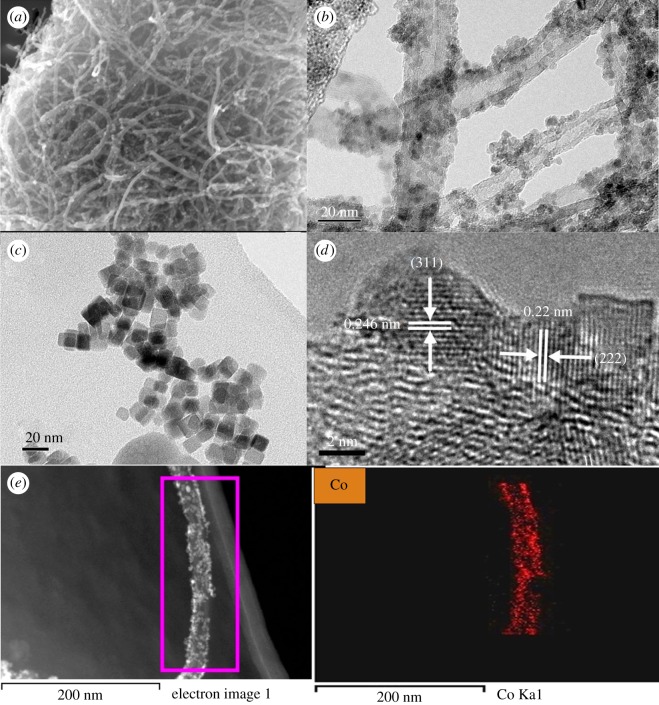
SEM image of Co_3_O_4_@MWNTs (*a*), TEM images of Co_3_O_4_@MWNTs (*b*) and Co_3_O_4_ (*c*), HRTEM image of Co_3_O_4_@MWNTs (*d*), energy dispersive X-ray spectroscopy spectrum (*e*) and distribution of Co atoms.

SEM image of the Co_3_O_4_@MWNTs composites consisted of 3D CNTs with uniformly attached Co_3_O_4_ nanoparticles (figure 2*a*), whereas SEM image of pure Co_3_O_4_ revealed a lump of powder with a disordered structure (electronic supplementary material, figure S1a). And the Co_3_O_4_ particles on Co_3_O_4_@MWNTs were 3–6 nm in size (figure 2*b*), smaller than that of the pure Co_3_O_4_ particles (figure 2*c*), which were about 8.5–10 nm. The rate of nucleation of Co_3_O_4_ was higher with the existence of MWNTs, leading to smaller particle size of Co_3_O_4_.

Typical HRTEM images of Co_3_O_4_@MWNTs showed lattice fringes with interplanar spacings of 0.22 and 0.246 nm, assignable to the (222) and (311) lattice planes of Co_3_O_4_, respectively (figure 2*d*), as with the pure Co_3_O_4_ (electronic supplementary material, figure S1b). These findings were consistent with the XRD results (figure 1*a*), indicating that Co_3_O_4_ had a good crystalline structure. The Co atoms showed a uniform distribution on the Co_3_O_4_@MWNTs (figure 2*e*), confirming that the Co_3_O_4_ particles were distributed equably on the surface of CNTs.

### 3.2. Reaction processes

**Figure 3.**
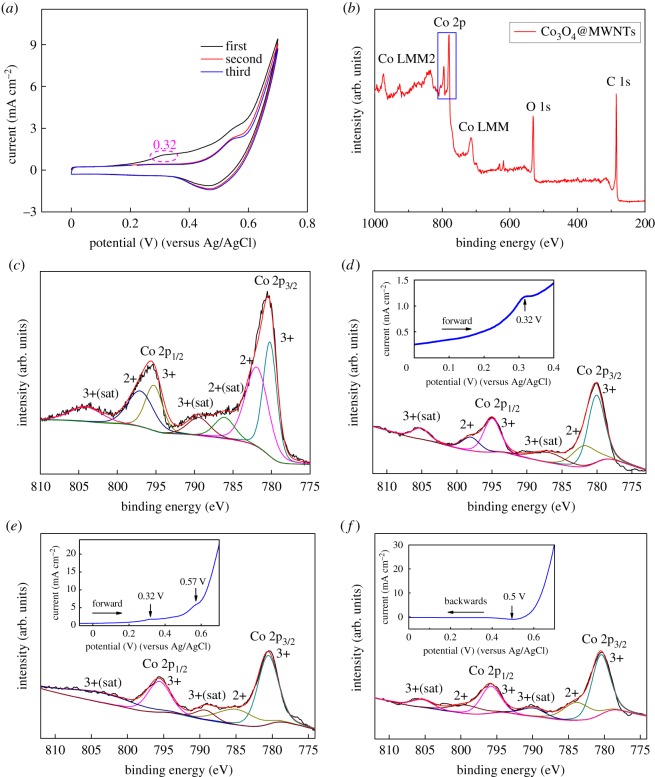
CV results of Co_3_O_4_@MWNTs in the first three cycles in 0.1 M KOH (*a*), survey XPS spectra of Co_3_O_4_@MWNTs (*b*), Co 2p core-levelled XPS spectrum of original Co_3_O_4_@MWNTs (*c*) and Co 2p core-levelled XPS spectrum of Co_3_O_4_@MWNTs after CV processes (0–0.4 V (*d*), 0–0.7 V (*e*) and 0.7–0 V (*f*)) shown in the insets, respectively.

To investigate the reaction mechanism of Co in the electrocatalysis, CV and XPS were conducted on Co_3_O_4_@MWNTs after different CV processes. We observed a pair of redox peaks at 0.57 V (oxidation) and 0.50 V (reduction). Interestingly, a weak oxidation peak appeared at about 0.32 V in the first cycle but disappeared in subsequent cycles (figure 3*a*).

The chemical composition of the Co_3_O_4_@MWNTs nanomaterial was detected by using XPS to characterize the Co oxidation state. Peaks corresponding to cobalt, oxygen and carbon were detected in the XPS spectra (figure 3*b*). The best deconvolution of the Co 2p profile was achieved with the assumption of seven species, including two pairs of spin-orbit doublets (indicating the coexistence of Co^2+^ and Co^3+^) and their three shakeup satellites (denoted as ‘sat’) [27]. The regional Co 2p spectrum revealed a high-energy band at 795.7 eV (Co 2p_1/2_) and a low-energy band at 780.4 eV (Co 2p_3/2_). These findings indicate the formation of Co_3_O_4_ on CNTs [28], as shown in figure 3*c*.

According to the CV results, the XPS measurement was conducted in three phases. Three identical electrodes were prepared as mentioned in the Electrochemical measurements section. CV experiments were performed at a scan rate of 1 mV s^−1^ with the three prepared electrodes as the working electrode in the range of 0–0.4, 0.4–0.7 and 0.7–0 V, respectively. Electrodes were removed from the electrolyte, cleaned with deionized water, dried at room temperature and subjected to XPS. The Co 2p core-levelled XPS spectrum of Co_3_O_4_@MWNTs and corresponding electrochemical processes are shown in figure 3*d–f*. When the CV of the original Co_3_O_4_@MWNTs was run to 0.32 V versus Ag/AgCl (inset of figure 3*d*), an oxidation peak appeared only in the first cycle. The ratio of Co^2+^ to Co^3+^ decreased from the Co 2p spectra in figure 3*d* compared to that in figure 3*c*, which indicates that Co^2+^ in Co_3_O_4_ was oxidized into Co^3+^ gradually in the electrochemical reaction. The reaction can be indexed to equation (3.1), as follows [13]:

3.1 $$Co_{3}O_{4}+OH^{-}+H_{2}O\to 3\mathrm{CoOOH}+e^{-}$$

As the reaction proceeded, an oxidation peak appeared at 0.57 V (inset of figure 3*e*), suggesting the oxidation of Co^3+^ into Co^4+^. This reaction can be indexed to the following equation (3.2) [13]:

3.2 $$\mathrm{CoOOH}+OH^{-}↔\mathrm{Co}O_{2}+H_{2}O+e^{-}$$

However, Co^4+^ species were not found on the Co 2p spectra in figure 3*e*, perhaps due to the rapid reduction of CoO_2_ into Co^3+^ in air.

When the potential reached 0.7 V, where Co_3_O_4_@MWNTs were adequately oxidized, the negative scan was started. A reduction peak was observed at 0.5 V versus Ag/AgCl (inset of figure 3*f*), corresponding to the reverse reaction of equation (3.2). After completion of the first cycle (inset of figure 3*f*), the XPS of the material was detected again. The resulting Co 2p spectrum in figure 3*f* was similar to that in figure 3*d* because the CoOOH experienced quasi-reversible oxidation and reduction and finally reformed CoOOH.

### 3.3. Electrochemical performance

**Figure 4.**
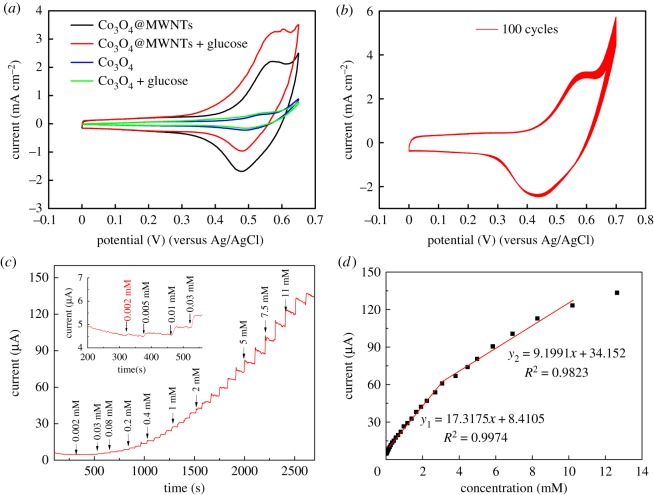
(*a*) CV results of Co_3_O_4_@MWNTs and Co_3_O_4_ in 0.1 M KOH with 3.0 mM glucose or without glucose. (*b*) CV of Co_3_O_4_@MWNTs for 100 cycles in 0.1 M KOH. (*c*) Typical amperometric response of Co_3_O_4_@MWNTs/GCE at 0.55 V to successive addition of glucose in 0.1 M KOH. (*d*) Calibration curve versus glucose concentration.

To investigate the electrochemical performance of the materials, CV measurements were conducted. Electronic supplementary material, figure S2 shows the CV results of Co_3_O_4_@MWNTs at different scan rates from 1 to 50 mV s^−1^. As scan rate decreased, the difference between the oxidation and reduction peaks (*Δ*Ep) decreased, indicating the quasi-reversible oxidation and reduction of the composite. CV results of Co_3_O_4_@MWNTs (black line) and Co_3_O_4_ (blue line) were obtained under the same conditions, with 0.1 M KOH as electrolyte, scan rate of 5 mV s^−1^ and voltage range of 0–0.65 V. We added 3 mM glucose solution to the electrolyte to study the electrochemical catalytic activity of the materials. The results were shown in figure 4*a*, where the current density of Co_3_O_4_@MWNTs was much higher than that of Co_3_O_4_, for two reasons: on the one hand, Co_3_O_4_@MWNTs have better conductivity than that of pure Co_3_O_4_; on the other hand, smaller size of Co_3_O_4_ particles in Co_3_O_4_@MWNTs leads to higher contact area with the electrolyte. In figure 4*a*, the current density of the electrode loading Co_3_O_4_ and Co_3_O_4_@MWNTs shifted overall in the positive direction after the addition of 3 mM glucose solution. This shift can be explained as follows: in the oxidation reaction, once the glucose solution was added, the oxidation product CoO_2_ near the anode would be consumed, as indicated by equation (3.3) [13]. Thus, the concentration of the reactant CoOOH would increase, resulting in an increase of the oxidation current density. Similarly, in the reduction reaction, once the glucose solution was added, the reactant CoO_2_ would decrease, resulting in a decrease of the reduction current density [13]. Thus, the observed shift of the current density in the positive direction indicates that Co_3_O_4_ catalysed the GOR.

3.3 $$2\mathrm{Co}O_{2}+C_{6}H_{12}O_{6}\to 2\mathrm{CoOOH}+C_{6}H_{10}O_{6}$$

The current density of the electrode loading the Co_3_O_4_@MWNTs had a stronger upward offset of 48.4% after 3 mM glucose was added to the electrolyte. This finding indicates that catalysis of the GOR by Co_3_O_4_@MWNTs was more effective than catalysis by pure Co_3_O_4_. The rapid reaction of CoO_2_ verified the phenomenon in figure 3*e* that there was no Co^4+^ observed, because CoO_2_ has a very strong oxidization and is reduced rapidly into Co^3+^. What is more, the cycle stability of the prepared Co_3_O_4_@MWNTs was detected by the CV for 100 cycles, as shown in figure 4*b*. There was nearly no decay with the curves, indicating a good cycle stability.

The real-time amperometric response of Co_3_O_4_@MWNTs to glucose was obtained by successive addition of increasing concentrations of glucose to the stirred 0.1 M KOH at 0.55 V (figure 4*c*). The fast response should be attributed to the contribution of the large surface of the Co_3_O_4_@MWNTs, excellent conductivity of CNTs, and good catalytic ability of the Co_3_O_4_ attached to the MWNTs. The electrode loading Co_3_O_4_@MWNTs responds linearly to glucose up to 5.8 mM, which can be used for the determination of glucose in blood samples (3–8 mM) [11]. The limit of detection is as low as 2 µM (S/N = 3). The linear range of the electrode loading Co_3_O_4_@MWNTs is up to 11 mM (correlation coefficient greater than 0.9823). Compared with some reported materials used for the determination of glucose, shown in table 1, Co_3_O_4_@MWNTs exhibit wider linear range, lower limit of determination and good sensitivity.

**Table 1.** The performance of different electrode materials used for glucose biosensor.

electrode material	sensitivity (μA cm^−2^ mM^−1^)	linear range (up to mM)	detection limit (μM)
graphene–Co_3_O_4_ [29]	11.9	0.3	10
3D-KSCs/hierarchical Co_3_O_4_ nanoclusters electrode [30]	1377	7	26
porous Au [31]	11.8	10	5.0
Cu*_x_*O/PPy/Au [32]	232.2	8	6.2
Ni/TiO_2_/Ti [33]	200.0	1.7	4
this work	239.0	11	2.0

### 3.4. Performance of the fuel cell

**Figure 5.**
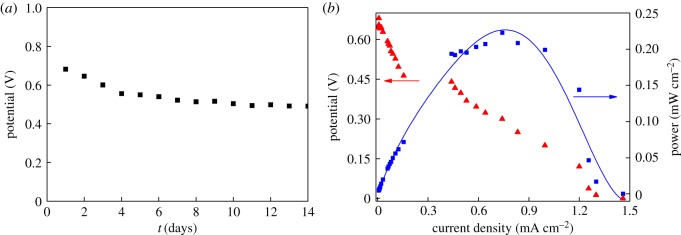
(*a*) The open circuit voltage from one cell over 15 days. (*b*) Voltage–current relation and power output of the fuel cell in the presence of 100 mM glucose. Blue line is the fitting of the power output.

A GFC was constructed with Co_3_O_4_@MWNTs as the anode material, due to its excellent ability to catalyse the GOR. As shown in figure 5*a*, our enzyme-free GFC is highly stable. The open circuit voltage drops only 27%. Figure 5*b* presents the voltage–current relation of the fuel cell with varying load resistance and corresponding power output, in the presence of 100 mM glucose. Some reported materials used for the glucose fuel cell were listed in table 2, from which the Co_3_O_4_@MWNTs showed higher open circuit voltage, short-circuit current density and power density. The glucose–O_2_ fuel cell exhibited an open circuit voltage of 0.68 V and short-circuit current density of 1.46 mA cm^−2^. The maximum power density of the glucose–O_2_ fuel cell was 0.22 mW cm^−2^ at 0.30 V.

**Table 2:** The performance of different electrode materials used for glucose fuel cell.

electrode material	open circuit voltage (V)	short-circuit current density (mA cm^−2^)	maximum power density (μW cm^−2^)
Au nanowire [6]	0.52	0.49	64
CNTs [34]	0.39	0.09	7.8
Pd nanowire [10]	0.25	0.41	72
Au nanowire [11]	0.43	1.34	126
this work	0.68	1.46	220

## 4. Conclusion

We successfully fabricated a high-performance nonenzymatic catalyst of Co_3_O_4_@MWNTs by a facile hydrothermal method. We studied the electrocatalytic mechanism of Co_3_O_4_@MWNTs through the state of the Co atoms. This mechanism can be explained as follows: Co_3_O_4_ are translated into CoOOH in KOH solution, and subsequently cycle between CoOOH and CoO_2_ in the electrochemical catalytic reactions. The Co_3_O_4_@MWNTs nanoarchitectures show high electrocatalytic activity for glucose oxidation. With its high open circuit voltage of 0.68** **V and short-circuit current density of 1.46** **mA cm^−2^, the Co_3_O_4_@MWNTs can be used as electrode materials in a GFC.

## Supplementary Material

Preparation of flow charts and partial electrochemical properties of Co3O4 @MWNTs

## Data accessibility

The datasets supporting this article have been uploaded as part of the electronic supplementary material.
