# Supplementary material for: Three-dimensional Co3O4@MWNTs nanocomposite with enhanced electrochemical performance for nonenzymatic glucose biosensors and biofuel cells
Source: R Soc Open Sci. 2017 Dec 20;4(12):170991. doi: 10.1098/rsos.170991 (PMC5750008; doi:10.1098/rsos.170991)
Supplement: Preparation of flow charts and partial electrochemical properties of Co3O4 @MWNTs [file rsos170991supp1.docx]

**Supporting Information**

**Three-dimensional Co_3_O_4_@MWNTs nanocomposite with enhanced electrochemical performance for nonenzymatic glucose biosensors and biofuel cells**

Kailong Jiao^a†^, Yu Jiang^c†^, Zepeng Kang^a^, Ruiyun Peng^b^, Shuqiang Jiao^a^, Zongqian Hu^b,^*

^a^ State Key Laboratory of Advanced Metallurgy, University of Science and Technology Beijing, Beijing, 100083, P.R. China.

^b^ Beijing Institute of Radiation Medicine, Beijing, 100850, P.R. China.

^c^ Department of Orthopedics, Peking University Third Hospital, Beijing, 100191, P.R. China.

^*^ To whom correspondence should be addressed. E-mail: huzongqian@hotmail.com (Z Hu); Tel: + 86-10-66930272

† These authors contributed equally to this work.

**Scheme S1** Schematic illustration of hydrothermal synthesis of the Co_3_O_4_@MWNTs in the enlarged view.

**Fig. S1** SEM image (a) and HRTEM image of Co_3_O_4_.

**Fig. S2** CVs of Co_3_O_4_@MWNTs with different scan rates.

**Fig. S3** N_2_ adsorption–desorption isotherms for the pure Co_3_O_4_ and Co_3_O_4_@MWNTs
